# Unusual Case of Recurrent Pneumothorax in Granulomatosis With Polyangiitis

**DOI:** 10.7759/cureus.24165

**Published:** 2022-04-15

**Authors:** Allison Franz, Sarmed Mansur

**Affiliations:** 1 Internal Medicine, The University of Toledo, Toledo, USA

**Keywords:** pulmonary fibrosis', diffuse alveolar hemorrhage, anca positive vasculitis, recurrent pneumothorax, granulomatosis with polyangiitis (gpa)

## Abstract

Granulomatosis with polyangiitis is a small vessel vasculitis that manifests as multisystemic inflammation predominantly affecting the lungs, upper respiratory tract, and the kidneys. Granulomatosis with polyangiitis commonly presents with elevated inflammatory markers and has a strong association with cytoplasmic antinuclear antibodies. Pulmonary manifestations of the disease include nodules, alveolar hemorrhage, and respiratory failure. The prevalence of pleural involvement is low, but can present as pleural effusion, wall thickening, and rarely pneumothorax. We describe the first report of recurrent pneumothorax secondary to presumed granulomatosis with polyangiitis.

## Introduction

Granulomatosis with polyangiitis (GPA) is a multisystemic inflammatory disease predominantly affecting the respiratory and renal systems that often presents with elevated inflammatory markers and has a strong association with antinuclear cytoplasmic antibodies [1,2]. Pulmonary GPA commonly presents as parenchymal cavitary nodules and rarely involves the pleural [1-3]. We describe a challenging case of recurrent pneumothorax likely secondary to granulomatosis with polyangiitis.

## Case presentation

**Figure 1:**
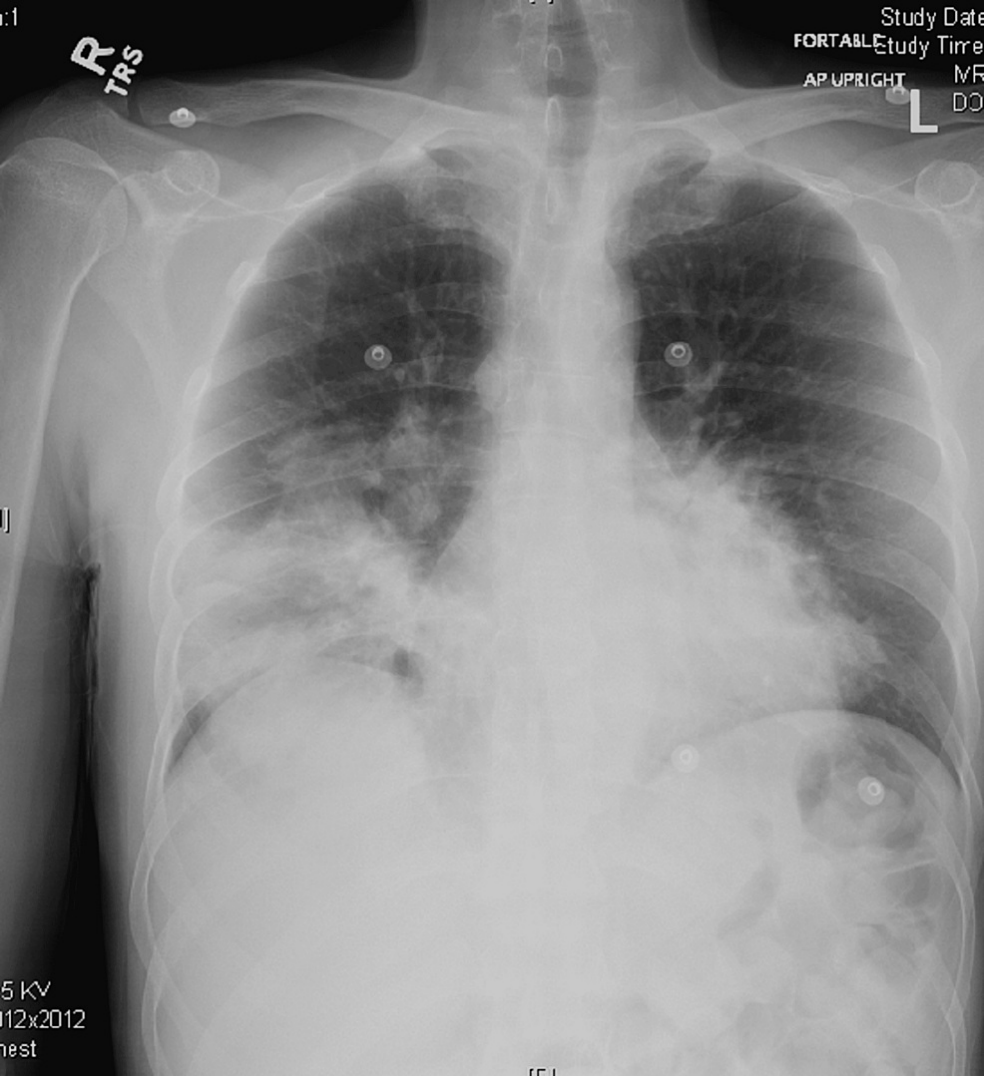
Portable anteroposterior chest radiograph on presentation showing right greater than left lung base airspace disease, confluent involving the right lung base.

A 46-year-old man without significant medical history was admitted to our institution for fatigue, cough, and fever. SARS-CoV-2-COVID-19 PCR was negative. Inflammatory markers included a C-reactive protein of 399 mg/L (nl: 0.00-7), procalcitonin of 0.35 ng/ml (nl: 0.00-0.10) and erythrocyte sedimentation rate of 130 mm/hr (nl: 0-10). Microbiological studies returned negative. Antineutrophil cytoplasmic antibody (ANCA) was positive at a titer of >1:1280 (reference range: <1:20) with a cytoplasmic pattern and specificity for serine protease 3 at a level of 907 AU/ml (reference range: 0-19). Anti-nuclear antibody was positive at 1:80 (reference range: <1/40). Serum creatinine was normal, and urinalysis showed microscopic hematuria. CBC was significant for mild eosinophilia of 6.8% (nl: 0.0-6.0) with an absolute eosinophil count 0.6 x 10^3^/ul (nl: 0.0-0.5). Imaging showed bilateral lower lobe airspace densities and large regions of confluent consolidations (Figure 1).

**Figure 2:**
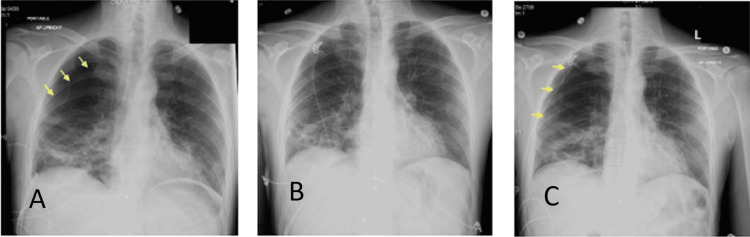
Portable anteroposterior chest radiographs showing A) initial right-sided pneumothorax, B) resolution of pneumothorax, C) recurrence of right-sided pneumothorax.

Without a definitive lung biopsy result, the presentation and laboratory studies supported a presumptive diagnosis of GPA with primarily pulmonary involvement. Initial hospital therapy included empiric broadspectrum antibiotics, intravenous immune globulin, high dose prednisone, and rituximab. He rapidly progressed into acute respiratory failure with hemoptysis, was transferred to the ICU, and intubated. Bronchoscopy showed diffuse alveolar hemorrhage. The patient remained intubated on mechanical ventilation for seven days due to poor respiratory status and hemodynamic instability. Following extubation, the patient was supported on supplemental oxygen and frequent incentive spirometry was encouraged. One month into his hospitalization, he developed new-onset chest pain, worsening dyspnea, and shortness of breath. He was found to have bilateral pneumothoraces and a small pulmonary embolism (Figure 2A). IV heparin was initiated as per nurse and then transition to apixaban and conservative pneumothorax treatment with 100% FiO2 nonrebreather were begun. Bilateral chest tubes were needed for complete lung re-expansion after three days of conservative treatment. A small air leak was noted from the right hemithorax. Eight days after initial development, chest X-ray (CXR) showed resolution of the bilateral pneumothoraces and chest tubes were removed (Figure 2B). Unfortunately, surveillance CXR five days later showed a recurrence of the right-sided pneumothorax (Figure 2C). The patient was having increased shortness of breath and increased oxygen requirement, an additional chest tube was placed and right-sided operative thoracoscopy with talc pleurodesis was performed with an ultimate resolution five days later.

## Discussion

Pneumothorax is a rare and potentially dangerous complication of granulomatosis with polyangiitis. In this case study, we describe the first report of recurrent pneumothorax in GPA and discuss potential contributing factors to initial pneumothorax and recurrence. The pathophysiology of pneumothorax in this patient population is unclear and several mechanisms have been proposed. Some have suggested pathogenic mechanisms that include the intrinsic activity of the primary disease, complicating infections, iatrogenic injuries, and the use of immunosuppressive therapy [4-7]. Storelli et al. suggest that pneumothorax is caused by spontaneous rupture of subpleural blebs and pulmonary bullae secondary to the fibrotic healing of active inflammation. Fibroelastosis and dense pleural fibrosis create traction on surrounding tissue leading to rupture of pulmonary blebs or bullae and the development of pneumothorax [6,8]. Our patient had elevated inflammatory markers indicating active disease on admission. Immunosuppressive therapy may contribute to this process by decreasing inflammation of the tissue and promoting healing through fibrosis. Furthermore, immunosuppressive therapy is associated with poor wound healing and increased rates of infection, hindering recovery from injuries due to pathologic and iatrogenic causes. Our patient was on a prolonged course of immunosuppressive therapy throughout his hospital course. Another potential contributing factor to pneumothorax is iatrogenic injury caused by frequent incentive spirometry and mechanical ventilation. Excessive use of incentive spirometry creates a negative intrathoracic pressure through inhalation that puts traction on the pleura and has been linked to pulmonary injury and development of barotrauma which may lead to rupture of subpleural blebs. Subsequently, fibrosis may decrease the compliance of the subpleural tissue making it more susceptible to repeated injury [9,10]. Factors that contributed to initial pneumothorax also likely played a role in recurrence. Our patient continued immunosuppressants and frequent incentive spirometry leading up to recurrence. Additionally, recurrence of pneumothorax is more common in patients with predisposing lung disease, a high height to weight ratio and treatment lacking pleurodesis [11].

## Conclusions

Pneumothorax is a rare occurrence in patients with GPA with no previous reports of recurrence. Our case is a novel case of recurrent pneumothorax five days after the resolution of initial pneumothorax. Recurrent pneumothorax in GPA with predominant pulmonary involvement should be considered when symptoms fail to improve as expected, especially in the settings of continued immune- and anti-inflammatory therapy and aggressive pulmonary toilet maneuvers including incentive spirometry.
